# Comparison of Flavonoid Compounds in the Flavedo and Juice of Two Pummelo Cultivars (*Citrus grandis* L. Osbeck) from Different Cultivation Regions in China

**DOI:** 10.3390/molecules191117314

**Published:** 2014-10-28

**Authors:** Mingxia Zhang, Haijuan Nan, Yanjie Wang, Xiaoying Jiang, Zheng Li

**Affiliations:** 1 Henan Institute of Science and Technology, Xinxiang 453003, Henan, China; E-Mails: nanhaijuan1@163.com (H.N.); mailyanjie@163.com (Y.W.); jxylxf@sohu.com (X.J.); 2 Food Science and Human Nutrition Department, Institute of Food and Agricultural Sciences, University of Florida, Gainesville, FL 32611, USA

**Keywords:** flavonoids, HPLC-MS, cluster analysis, cultivation regions

## Abstract

The objective of this study was to investigate the effect of different cultivation regions on the pattern and content of flavonoids in two pummelo cultivars (*C. grandis* L. Osbeck) in China. Results showed that similar patterns of flavonoids were observed in the flavedo or juice of each pummelo cultivar from these cultivation regions, whereas the individual flavonoid content showed unique characteristics. Naringin, the predominant flavanone glycoside, showed the highest content in both flavedo and juice of *C. grandis* “Guanximiyu” from the Pinghe of Fujian (FJ) cultivation region compared with the Dapu of Guangdong (GD) and Nanbu of Sichuan (SC) regions. However, its content in the flavedo of *C. grandis* “Shatianyu” from the Pingle of Guangxi (GX) was significantly lower than in the GD and SC regions. Vicenin-2 appeared to be the dominant flavone *C*-glycoside in the flavedo of both cultivars, and the lowest content was observed in the flavedo of *C. grandis* “Guanximiyu” from the SC region. However, *C. grandis* “Shatianyu” contained the highest content of vicenin-2 in the flavedo from SC region. Similarly, the predominant flavone *O*-glucoside, rhoifolin, showed the highest content in *C. grandis* “Guanximiyu” from the GD and FJ regions, whereas *C. grandis* “Shatianyu” in SC region showed the highest content of rhoifolin. Cluster analysis suggested that genotype played a primary role in determining the flavonoid profiles of pummelo cultivars, whereas regional differences significantly affected the flavonoid distribution of pummelo cultivars potentially via affecting the direction of flavonoid accumulation in pummelo.

## 1. Introduction

Phenolic compounds have received considerable attention in the food science field because they have been demonstrated to possess multiple health benefits [1,2,3,4]. Phenolic compounds naturally exist as secondary metabolites in fruits, and their accumulation is determined by genotypes as well as regional cultivation conditions [5,6,7]. For a particular variety of fruit, different regional cultivation characteristics have been reported to regulate the flavonoid metabolism gene expressions and enzyme activities, and therefore play a very important role in affecting the accumulation of phenolic compounds [8,9]. Therefore, understanding the impact of the cultivation regions on the pattern and content of phenolic compounds in pummelo is useful for the cultivation of pummelo of better quality and nutritional value.

Pummelo (*C. grandis* L. Osbeck) belongs to the Citrus genus and is widely cultivated in the southern areas of China [10]. *C. grandis* “Guanximiyu” and “shatianyu” are two major pummelo cultivars that are widely cultivated in China. Previous studies have confirmed that pummelo contains high levels of phenolic compounds, especially flavonoids, such as flavanones and flavones [11,12,13]. However, whether the pattern and content of flavonoids in a single pummelo cultivar are affected by cultivation region has so far not been investigated in China. Therefore, we chose two pummelo cultivars, *C. grandis* “Guanximiyu” from Dapu of Guangdong (GD), Pinghe of Fujian (FJ), and Nanbu of Sichuan (SC), and *C. grandis* “Shatianyu” from Dapu of Guangdong (GD), Pingle of Guangxi (GX), and Nanbu of Sichuan (SC) of China. *C. grandis* “Guangximiyu” was originally cultivated in the FJ region, whereas the GX region is the original cultivation area for *C. grandis* “Shatianyu”. The pattern and composition of the flavonoids from these regional pummelo cultivars were analyzed using high performance liquid chromatography coupled with mass spectrometry (HPLC-MS/MS). The objective of this study was to investigate the effects of cultivation regions on flavonoid pattern and distribution in the flavedo and juice of pummelo cultivars in China, and further to test whether the flavonoid composition could be used as an indicator to differentiate the regional effects.

## 2. Results

### 2.1. Comparison of Flavonoids in C. grandis “Guanximiyu” Flavedo

A total of five flavanone glycosides were detected in the flavedo of *C. grandis* “Guanximiyu” (Table 1). The total content of flavanone glycosides in the flavedo of *C. grandis* “Guanximiyu” was the highest in the FJ cultivation region, followed by the GD and SC regions. Naringin was the major flavanone glycoside in *C. grandis* “Guanximiyu” flavedo from all the cultivation regions, followed by *O*-triglycosylnaringenin and acetylnaringin. Neoeriocitrin and melitidin were present in the flavedo samples as well. However, their contents appeared to be at a low level. FJ flavedo showed the highest naringin content compared to the flavedo from the other two cultivation regions. Similarly, the other three flavanone glycosides in the flavedo of *C. grandis* “Guanximiyu”, including *O*-triglycosyl- naringenin, acetylnaringin, and neoeriocitrin, showed the highest content in the FJ cultivation region. However, no significant differences in the content of melitidin in the flavedo were observed from these cultivation regions.

**Table 1:** Content of individual flavonoids in *C. grandis* “Guanximiyu” flavedo from different cultivation regions.

Flavonoid	Flavedo (mg/kg Fresh Weight)		
	Dapu of Guangdong	Pinghe of Fujian	Nanbu of Sichuan
	(GD)	(FJ)	(SC)
***Flavanone glycoside***			
*O*-Triglycosylnaringenin	353.55 ± 72.78 ^b^	535.17 ± 20.23 ^a^	410.65 ± 14.73 ^b^
Neoeriocitrin	57.02 ± 11.62 ^b^	75.31 ± 9.14 ^a^	50.61 ± 1.49 ^b^
Naringin	3594.62 ± 470.90 ^b^	5084.29 ± 484.88 ^a^	2458.34 ± 63.33 ^c^
Acetylnaringin	202.89 ± 43.01 ^b^	263.09 ± 26.81 ^a^	209.73 ± 5.04 ^b^
Melitidin	57.32 ± 27.81 ^a^	73.88 ± 6.24 ^a^	55.97 ± 7.13 ^a^
*Total flavanone glycoside*	4265.40	6031.74	3185.30
***Flavone C-glycoside***			
Lucenin-2	15.14 ± 6.04 ^b^	26.96 ± 3.71 ^a^	23.04 ± 5.29 ^a^
Vicenin-2	401.95 ± 79.33 ^a^	351.35 ± 52.80 ^a,b^	200.26 ± 32.52 ^b^
Apigenin 6-*C*-glucosyl-7-*O*-glucoside	28.63 ± 7.14 ^a^	20.45 ± 3.70 ^b^	12.34± 2.41 ^c^
Lucenin-2 4'-methyl ether	--	24.81 ± 6.27 ^a^	15.62 ± 2.89 ^a^
Diosmetin 6-*C*-glucoside	--	39.19 ± 4.10 ^a^	29.95 ± 14.03 ^a^
Apigenin 6,8-di-*C*-(sinapolyl) glycoside	72.50 ± 16.60 ^a^	73.40 ± 11.28 ^a^	63.94 ± 9.52 ^a^
Apigenin 6,8-di-*C*-(feryloyl) glycoside	56.74 ± 13.73 ^b^	73.96 ± 7.36 ^a^	56.56 ± 8.69 ^b^
*Total flavone C-glycoside*	574.96	610.12	401.71
***Flavone O-glucoside***			
Kaempferol 7-*O*-rhamnosyl 3-*O*-glucoside	23.19 ± 7.75 ^a^	26.82 ± 2.52 ^a^	30.23 ± 4.80 ^a^
Rhoifolin	429.32 ± 59.51 ^a^	339.62 ± 26.94 ^a^	166.93 ± 20.34 ^b^
Diosmin	38.74 ± 7.43 ^a,b^	46.01 ± 3.52 ^a^	23.06 ± 12.41 ^b^
*Total flavone O-glucoside*	491.25	412.45	220.22

-- represents not detected; data are mean ± standard deviation of triplicate tests from three trees; different letters in each compound represent significant difference at *p* not det.

Seven flavone *C*-glycosides were detected in the flavedo of *C. grandis* “Guanximiyu” from these cultivation regions, although neither lucenin-2,4'-methyl ether nor diosmetin 6-*C*-glucoside were found in the GD flavedo sample. The total content level of flavone *C*-glycosides in the flavedo of *C. grandis* “Guanximiyu” was comparable with that in the GD and FJ regions. The total content of flavone *C*-glycosides was approximately 1.5 times higher in the FJ than in the SC region. With regards to individual flavone *C*-glycosides, the predominant flavone *C*-glycoside in the flavedo of *C. grandis* “Guanximiyu” appeared to be vicenin-2 and its content showed a regional difference. The GD and FJ regional flavedo samples contained a higher level of vicenin-2 than the SC flavedo. It should be noted that apigenin 6-*C*-glucosyl-7-*O*-glucoside was not predominantly present in the flavedo of *C. grandis* “Guanximiyu”. However, its content showed the regional differences. Its content was higher in the GD cultivation region, followed by the FJ and SC. The FJ and SC cultivation regions showed higher content levels of lucenin-2 and apigenin 6,8-di-*C*-(feryloyl) glycoside in comparison with the GD region. No significant differences in the content of other flavone *C*-glycosides were observed in the flavedo samples from these cultivation areas.

Three individual flavone *O*-glucosides were identified in the flavedo of *C. grandis* “Guanximiyu” from all the cultivation regions. However, their content levels showed significant differences. The total content of flavone *O*-glucosides appeared to be the lowest in the SC region and the content from the GD and FJ regions was approximately two times higher than in SC. The significant differences of total flavone *O*-glucosides resulted from rhoifolin, the predominant flavone *O*-glycoside. The GD and FJ regions had approximately 2–3 times higher content of rhoifolin in comparison with the SC cultivation region. Similarly, diosmin showed the highest content level in the FJ cultivation region followed by the GD and SC. A similar content level of kaempferol 7-*O*-rhamnosyl 3-*O*-glucoside was observed in the flavedo of *C. granids* “Guanximiyu” from all the cultivation regions.

### 2.2. Comparison of Flavonoids in C. grandis “Guanximiyu” Juice

In the juice of *C. grandis* “Guanximiyu”, six individual flavanone glycosides were detected. The GD regional juice did not contain *O*-triglycosyl naringenin or neoeriocitrin, although the content level of these two flavanone glycosides was low in the juice from the FJ and SC regions (Table 2). The total contents of flavanone glycosides showed similar results as the flavedo samples, and the FJ and GD cultivation regions showed approximately two times higher total content compared with the SC region. Regarding individual flavanone glycosides, naringin also appeared to be the predominant flavanone glycoside. Its content in the juice of *C. grandis* “Guanximiyu” from the GD and FJ cultivation regions was approximately two times higher than that in the SC juice sample. This trend was also observed in acetyl naringin content in the juice sample from these regions. *C. grandis* “Guanximiyu” juice in the FJ cultivation region showed the highest melitidin content, followed by the GD and SC regions. It should be observed that *O*-triglycosylnaringenin, neoeriocitrin, and didymin existed at a relatively low level in all the juice samples except that *O*-triglycosylnarigenin and neoeriocitrin were not present in the GD region.

The only flavone *C*-glycoside detected in the juice of *C. grandis* “Guanximiyu” was vicemin-2, and it was found at a relatively low level, while the FJ region juice sample appeared to have the highest content.

There were two flavone *O*-glucosides in the juice of *C. grandis* “Guanximiyu” from all the cultivation regions. Interestingly, rhoifolin turned out to be the predominant flavone *O*-glucoside, and the primary contributor to total flavone *O*-glucoside content. Its content in the juice of *C. grandis* “Guanximiyu” from the GD and FJ regions was approximately three times higher than that in the SC region. Diosmin also showed significantly different levels in these regional juices, although its content was low.

**Table 2:** Content of individual flavonoids in *C. grandis* “Guanximiyu” juice from different cultivation regions.

Flavonoid	Juice (mg/L)		
	Dapu of Guangdong	Pinghe of Fujian	Nanbu of Sichuan
	(GD)	(FJ)	(SC)
***Flavanone glycoside***			
*O*-Triglycosylnaringenin	--	1.32 ± 0.11 ^a^	0.75 ± 0.58 ^a^
Neoeriocitrin	--	1.39 ± 0.14 ^a^	0.80 ± 0.09 ^b^
Didymin	0.35 ± 0.03 ^a^	0.19 ± 0.02 ^b^	0.26 ± 0.04 ^b^
Naringin	121.42 ± 4.84 ^a^	139.21 ± 15.00 ^a^	60.98 ± 6.55 ^b^
Acetylnaringin	3.27 ± 0.17 ^b^	4.95 ± 0.50 ^a^	2.22 ± 0.24 ^b^
Melitidin	8.77 ± 0.46 ^b^	12.85 ± 1.11 ^a^	4.41 ± 0.19 ^c^
*Total flavanone glycoside*	133.81	159.91	69.42
***Flavone C-glycoside***			
Vicenin-2	0.43 ± 0.15 ^b^	0.82 ± 0.25 ^a^	0.56 ± 0.44 ^b^
*Total flavone C-glycoside*	0.43	0.82	0.56
***Flavone O-glucoside***			
Rhoifolin	20.02 ± 0.94 ^a^	23.24 ± 2.31 ^a^	7.40 ± 0.81 ^b^
Diosmin	0.34 ± 0.04 ^b^	0.74 ± 0.06 ^a^	0.16 ± 0.02 ^c^
*Total flavone O-glucoside*	20.36	23.98	7.56

-- represents not detected; data are mean ± standard deviation of triplicate tests from three trees; different letters in each compound represent significant difference at *p* represe.

### 2.3. Comparison of Flavonoids in C. grandis “Shatianyu” Flavedo

Except for neohesperidin being only present in the flavedo of *C. grandis* “Shatianyu” from the GX region, the flavedo samples from the GD, GX, and SC cultivation regions all contained five flavanone glycosides. However, the content of these flavanone glycosides showed regional characteristics (Table 3). Total content of flavanone glycosides showed a higher level in both GD and SC cultivation regions, and the content was approximately two times higher than that in the GX flavedo sample. According to individual flavanone glycosides, naringin and melitidin were two predominant flavanone glycosides present in the flavedo of *C. grandis* “Shatianyu”, and the GD and SC flavedo samples contained significantly higher content of naringin and melitidin than the flavedo sample from the GX region. It should also be noted that the content of naringin in the SC flavedo was approximately five times higher than that in the GX, whereas *C. grandis* “Shatianyu” flavedos in the GD and SC had almost 1.5–2.0 fold the content of melitidin compared with the GX flavedo sample. The content of *O*-triglycosyl naringenin and acetyl naringin turned out to be the highest in the GD region followed by the SC and GX. Neoeriocitrin had a similar content level in these regional flavedo samples but differences among regions were not statistically significant.

Vicenin-2 and apigenin 6,8-di-*C*-(sinapolyl) glycoside were two flavone *C*-glycosides detected in the flavedo of *C. grandis* “Shatianyu” from these cultivation regions. However, apigenin 6,8-di-*C*-(feryloyl) glycoside was detected only in the SC flavedo sample at a high content level. The SC cultivation region resulted in the highest total content of flavone *O*-glycoside in comparison with the flavedo sample from the GD and GX regions. In comparison to vicenin-2 in the regional flavedo samples, the highest content was observed in the SC region, followed by the GD and GX regions. Interestingly, The GD and GX *C. grandis* “Shatianyu” flavedos had similar content levels of apigenin 6,8-di-*C*-(sinapoly) glycoside, which were higher than the level in the cultivar from the SC region.

The flavedo of *C. grandis* “Shatianyu” from the GD, GX, and SC regions all contained rhoifolin, a flavone *O*-glucoside. However, the content showed significant differences according to the region. The SC regional *C. grandis* “Shatianyu” flavedo had the highest rhoifolin content, which was about two and five times higher than the GD and GX flavedo, respectively. Diosmin was only detected in the flavedo of *C. grandis* “Shatianyu” pummelo cultivated in the SC region.

**Table 3:** Content of individual flavonoids in *C. grandis* “Shatianyu” flavedo from different cultivation regions.

Flavonoid	Flavedo (mg/kg Fresh Weight)		
	Dapu of Guangdong	Pingle of Guangxi	Nanbu of Sichuan
	(GD)	(GX)	(SC)
***Flavanone glycoside***			
*O*-Triglycosylnaringenin	193.26 ± 32.68 ^a^	129.38 ± 3.73 ^b^	126.37 ± 7.75 ^b^
Neoeriocitrin	18.53 ± 2.94 ^a^	16.11 ± 0.74 ^a^	14.71 ± 3.75 ^a^
Naringin	708.91 ± 22.70 ^b^	220.22 ± 15.93 ^c^	987.85 ± 6.87 ^a^
Neohesperidin	--	29.15 ± 4.13	--
Acetyl naringin	72.50 ± 8.87 ^a^	50.76 ± 5.56 ^b^	55.68 ± 3.13 ^b^
Melitidin	354.72 ± 10.71 ^b^	203.53 ± 15.09 ^c^	466.48 ± 21.83 ^a^
*Total flavanone glycoside*	1347.92	649.15	1650.99
***Flavone C-glycoside***			
Vicenin-2	62.92 ± 5.31 ^b^	34.48 ± 3.53 ^b^	125.63 ± 11.20 ^a^
Apigenin 6,8-di-*C*-(sinapolyl) glycoside	54.58 ± 1.63 ^a^	57.98 ± 2.55 ^a^	36.63 ± 3.57 ^b^
Apigenin 6,8-di-*C*-(feryloyl) glycoside	--	--	40.53 ± 2.23
*Total flavone C-glycoside*	117.50	92.46	202.79
***Flavone O-glucoside***			
Rhoifolin	37.40 ± 13.95 ^b^	14.19 ± 1.32 ^c^	74.23 ± 41.56 ^a^
Diosmin	--	--	13.89 ± 0.51
*Total flavone O-glucoside*	37.40	14.19	88.12

-- represents not detected; data are mean ± standard deviation of triplicate tests from three trees; different letters in each compound represent significant difference at *p* ≤ 0.05.

### 2.4. Comparison of Flavonoids in C. grandis “Shatianyu” Juice

In the juice of *C. grandis* “Shatianyu”, except for neoeriocitrin being detected in only GX regional juice, the other three flavanone glycosides, including naringin, acetylnaringin, and melitidin were all present in the juice samples from these cultivation regions (Table 4). The total contents of flavanone glycosides were similar in the juices from these cultivation regions. Naringin and melitidin were two major flavanone glycosides that existed in the juice of *C. grandis* “Shatianyu”. However, no significant differences in their content were observed among these cultivation regions. It should be noted that acetylnaringin content was much higher in the juice from the SC region compared with the GD and GX, although the content level of acetylnaringin was significantly low in the juice.

Rhoifolin was the only flavone *O*-glucoside detected in all the regional *C. grandis* “Shatianyu” juices, and the SC regional juice turned out to have the highest content followed by the juice samples from the GD and GX cultivation regions.

**Table 4:** Content of individual flavonoids in *C. grandis* “Shatianyu” juice from different cultivation regions.

Flavonoid	Juice (mg/L)		
	Dapu of Guangdong	Pingle of Guangxi	Nanbu of Sichuan©
	(GD)	(GX)	(SC)
***Flavanone glycoside***			
Neoeriocitrin	--	2.36 ± 0.48	--
Naringin	198.52 ± 18.10 ^a^	164.33 ± 16.31 ^a^	175.90 ± 18.26 ^a^
Acetylnaringin	2.43 ± 0.04 ^b^	2.45 ± 0.12 ^b^	4.70 ± 0.37 ^a^
Melitidin	274.89 ± 17.25 ^a^	218.86 ± 29.55 ^a^	258.08 ± 31.73 ^a^
*Total flavanone glycoside*	475.84	388.00	438.68
***Flavone O-glucoside***			
Rhoifolin	10.08 ± 1.28 ^a,b^	8.32 ± 0.89 ^b^	17.34 ± 1.30 ^a^
*Total flavone O-glucoside*	10.08	8.32	17.34

-- represents not detected; data are mean ± standard deviation of triplicate tests from three trees; different letters in each compound represent significant difference at *p* ≤ 0.05.

### 2.5. Cluster Analysis

**Figure 1:**
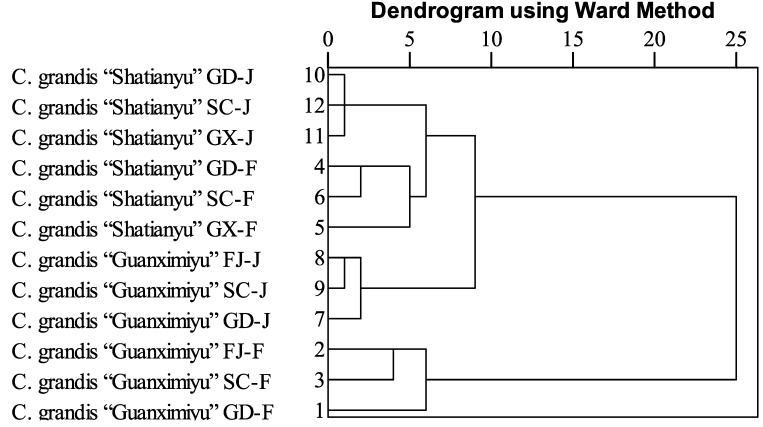
The hierarchical cluster of flavonoids in pummelo (*C. grandis* “Guanximiyu” and *C. grandis* “Shatianyu”) flavedo and juice from different cultivation regions. GD, FJ, GX, SC represent the region of Dapu of Guandong, Pinghe of Fujian, Pingle of Guangxi, and Nanbu of Sichuan, respectively. F and J represent flavedo and juice, respectively.

Cluster analysis was carried out using all the flavonoids quantified as variables with the aim of better understanding the similarities and differences of flavonoid profiles in the flavedo and the juice of these two pummelo cultivars from different cultivation regions in China (Figure 1). The proximity among samples was determined by the squared Eulidean distance using Ward’s method as a linkage rule. It should be noted that the original cultivation regions of *C. grandis* “Guanximiyu” and “Shatianyu” are FJ and GX, respectively. Regarding the flavonoid profile in the juice of *C. grandis* “Guanximiyu”, FJ and SC regions were clustered with a closer hierarchical distance in comparison with the GD region, indicating that the flavonoid profiles from FJ and SC regions were quite similar. Similarly, the same hierarchical distances were observed in the juice of *C. grandis* “Shatianyu” from GD, GX, and SC regions, suggesting they possessed the similar flavonoid profile in the juice. Compared to the flavonoid profile in the juice of pummelo cultivars, much more differences were observed in the flavedo of pummelo cultivars from different cultivation regions. For example, in the flavedo of *C. grandis* “Guanximiyu”, FJ and SC showed a much closer hierarchical distance compared with GD, indicating that the GD cultivation region significantly changed the flavonoid profile in the flavedo of *C. grandis* “Guanximiyu”, and its flavonoid profile was no longer similar to that from its original cultivation region, FJ. Similarly, the flavedo of *C. grandis* “Shatiamyu” from GD and SC regions shared a similar flavonoid profile, and the profile was significantly different from the GX region, the original cultivation area of *C. grandis* “Shatianyu” in China. These results indicated that cultivation regions with different regional characteristics played an important role in the accumulation of flavonoids in pummelo cultivars and significantly changed the flavonoid profile of each pummelo cultivar, especially the flavonoid profile of the pummelo flavedo.

## 3. Discussion

Different flavonoid patterns in both flavedo and juices of these two *C. grandis* pummelo cultivars were observed, suggesting that the biosynthesis of flavonoids in pummelo was primarily determined by pummelo genotypes [13,14]. However, the pattern of the flavonoids in the flavedo or the juice of each pummelo cultivar was quite similar from different cultivation regions, whereas the content of individual flavonoids appeared to be significantly different. These results indicated that cultivation region significantly affected the accumulation direction of flavonoids in the flavedo and the juice of pummelos [8,15,16]. It has been proposed that the expressions of genes and activities of enzymes related to flavonoid biosynthesis and metabolism can be regulated and altered in different cultivation regions under different temperatures, precipitations, and sunshine exposures, which eventually impact the accumulation of the flavonoids in fruits [17,18]. In the biosynthesis of flavonoids in fruits, L-phenylalanine is catalyzed by a series of enzymes to form 4-coumaroyl CoA. Subsequently, naringenin chalcone is biosynthesized from 4-coumaroyl CoA and malonyl CoA with the activity of chalcone synthase (CHS). With the presence of chalcone isomerase (CHI), naringenin chalcone is further converted into naringenin. Finally, naringenin derivatives are yielded from naringenin during a series of enzymatic reactions by conjugating different sugar moieties [1,19,20,21]. Thus, naringenin and its derivatives can be used as an indicator to reflect the impact of cultivation regions in gene expression and enzyme activity levels of flavonoid biosynthesis. In the present study, the highest total flavanone glycosides in *C. grandis* “Guanximiyu” flavedo and juice were observed in FJ and GD regions. These results suggested that CHS and CHI genes might show a level of higher expression in these two regions, increasing the activity of CHS and CHI to enhance the accumulation of flavanone glycosides in the flavedo of *C. grandis* “Guanximiyu”. Regarding the regional characteristics of these cultivation regions (Table 5), the FJ and GD regions have much higher average annual precipitation and temperature in comparison with the SC cultivation area. These regional characteristics may help to stimulate the expression of CHS and CHI genes in pummelo, leading to higher contents of flavanone glycosides. Surprisingly, the original cultivation regions of *C. grandis* “Shatianyu”, the GX region, had the lowest level of flavanone glycosides in both flavedo and juices in comparison with the GD and SC regions. Although the GD and GX showed similar climates as well as regional characteristics, the microclimate in the GD region might be an important factor that caused the higher accumulation of flavanone glycosides by potentially increasing CHS and CHI enzyme activities in *C. grandis* “Shatianyu”. Interestingly, the ratios of naringin to the total flavanone glycosides in *C. grandis* “Guanximiyu” remained similar when comparing the FJ region with the GD and SC regions, suggesting that the expression of genes that regulate conjugation of sugar moieties to naringenin remained similar under different cultivation regions. However, obviously low ratios of naringin to total flavanone glycosides in the flavedo of *C. grandis* “Shatianyu” from the GX region indicated that the expression of the glycosyltransferase gene might be significantly regulated by the GX regional characteristics.

Naringenin forms apigenin under the catalytic activity of flavone synthase I (FNS I) and flavone synthase II (FNS II). Naringenin is also catalyzed by flavanone-3-hydroxylase (FHT) to yield dihydrokaempferol which is further converted into kaempferol under the activity of flavonol synthase (FLS). Afterwards, flavones are conjugated with sugar moieties to form flavone *C*-glycosides and flavone *O*-glucosides via *C*-glucosyltransferase and *O*-glucosyltransferase, respectively [22,23,24]. Therefore, the level of apigenin and its derivatives is considered an indicator for evaluating the regulation of flavone synthase genes by cultivation regions. In the present study, apigenin and its derivatives were most observed in the flavedo of *C. grandis* “Guanximiyu” and “Shatianyu” cultivars, whereas the juices did not contain high levels of apigenin and its derivatives. In *C. grandis* “Guanximiyu” flavedo, the total contents of flavone *C*-glycosides and *O*-glucosides were much higher in the FJ and GD regions, indicating that the FNS I and FNS II might show higher activity from these two cultivation regions. In the flavedo of *C. grandis* “Shatianyu” cultivar, the lower level of flavone *C*-glycosides and *O*-glucosides from the GX region suggested the lower activity of flavone synthases caused by low expression of the flavone synthase genes. With regards to individual flavones, vicenin-2 and apigenin 6,8-di-*C*-β-d-glucopyranoside were the predominant apigenin derivatives. The different content ratio in each cultivar from different regions indicated that the *C*-glucosyltransferase metabolisms were not consistent in different cultivation regions. It should also be noticed that rhoifolin and apigenin 7-*O*-neohesperidoside were the major flavone *O*-glucosides in the flavedo and juices of these two pummelos. However, the content level of rhoifolin showed the cultivation region characteristics, indicating that *O*-glucosyltransferase enzyme activities were very different in these cultivation regions, which might be due to different *O*-glucosyltransferase gene expression levels in these cultivation regions.

**Table 5:** Location, climate, and regional characteristics of different pummelo cultivation regions in China.

Cultivation Region	Location	Climate	Regional Characteristics	Soil Type
Dapu of Guangdong (GD)	Dapu of Guangdong province, Northeastern area of Guangdong province, upper- and mid- stream of Hanjiang River, mountains within the region and central area filled with hills	Subtropical monsoon	North Latitude 24°35', East Longitude 116°59'; Average annual precipitation 1400 to 1800 mm; Average annual temperature 20.7 to 21.4 °C; Average annual sunshine over 2000 h	Red earths
Pingle of Guangxi (GX)	Pingle of Guangxi province, Northeastern area of Guanxi province, merging area of Lijiang, Li Jiang, and Chajiang Rivers, Karst landform characteristics	Subtropical monsoon	North Latitude 24°37', East Longitude 110°40', Elevation 104 m; Average annual precipitation 1355 to 1865 mm; Average annual temperature 19.9 °C; Average annual sunshine 1414 to 2094 h	Acid purplish soils
Pinghe of Fujian (FJ)	Pinghe of Fujian province, Southwestern of Fujian Golden Triangle in Fujian province, connected with Guangdong province	Subtropical monsoon	North Latitude 24°02' to 24°35', East Longitude 116°54' to 117°31'; Average annual precipitation 1724 mm; Average annual temperature 17.5 to 21.3 °C; Average annual sunshine 1758 to 1852 h	Lateritic red earths
Nanbu of Sichuan (SC)	Nanbu of Sichuan province, Northeastern Sichuan Basin, midstream of Jialing River, covered with Qinling and Daba mountains	Subtropical humid monsoon	North Latitude 31°04' to 31°40', East Longitude 105°27' to 106°24'; Average annual precipitation 970 mm; Average annual temperature 16.8 °C; Average annual sunshine 1296 h	Calcic purplish soils

The original cultivation region of *C. grandis* “Guanximiyu” and “Shatianyu” cultivar is Pinghe of Fujian (FJ) and Pingle of Guangxi (GX), respectively.

Geographically, The GD and GX regions belong to the south China cultivation area for pummelo, whereas the FJ and SC region are located in the southeast and southwest pummelo cultivation area, respectively. The GD and GX regions possess a subtropical monsoon climate with average annual precipitation approximately 1500 nm, average annual temperature approximately 20 °C, and average annual sunshine of approximately 2000 h (Table 5). The FJ region possesses similar climate characteristics compared to the GD and GX regions. It should be noted that the SC region is located in the Sichuan basin, and has a subtropical humid monsoon climate. Its average annual precipitation is much lower than the GD, GX, and FJ regions [10]. From the cluster analysis (Figure 1), the profiles of flavonoids in *C. grandis* “Guanximiyu” juice were quite similar among GD, FJ and SC cultivation regions. The similar profiles of flavonoids in *C. grandis* “Shatianyu” juices from GD, GX, and SC regions were also observed. However, the flavonoid profiles in the flavedo of these two cultivars from these cultivation regions showed much greater differences. Regarding *C. grandis* “Guanximiyu”, the FJ and SC cultivation regions showed a much closer flavonoid profile compared to GD region, which might be due to the difference of cultivation regions. However, it should be observed that the GD region shared a similar flavonoid profile with the SC region, instead of the GX region in the flavedo of *C. grandis* “Shatianyu”. This indicated that the microclimate of the GD region might significantly alter the accumulation of flavonoids. More importantly, the better flavonoid profiles in the flavedo of *C. grandis* “Shatianyu” cultivar were observed in the GD and SC regions rather than its original cultivation region, GX.

## 4. Experimental Section

### 4.1. Chemicals and Standards

Methanol, acetic acid, and acetonitrile were purchased from Fisher Scientific (Fairlawn, NJ, USA). The analytical grade ethyl acetate was received from the Beijing Chemical Reagent Plant (Beijing, China). Naringin and quercitrin were purchased from Extrasynthese SA (Genay, France). Millipore water was generated from a Milli-Q Element water purification system (Millipore, Bedford, MA, USA).

### 4.2. Fruits

*C. grandis* “Guanximiyu” from Dapu of Guangdong (GD), Pinghe of Fujian (FJ), and Nanbu of Sichuan (SC), and *C. grandis* “Shatianyu” from Dapu of Guangdong (GD), Pingle of Guangxi (GX), and Nanbu of Sichuan (SC) were harvested between September and November of 2009. Three fully ripened fruits were randomly picked in the same tree and in each genotype. Three trees were randomly selected in the same orchard. The regional characteristics of these cultivation regions are listed in Table 5. The physiochemical properties of each pummelo cultivar from the different cultivation regions were similar (Table 6).

**Table 6:** Total sugar, total acid, and soluble solid content of *C. grandis* “Guanximiyu” in GD, FJ, and SC, and *C. grandis* “Shatianyu” in GD, GX, and SC, respectively.

Cultivation Region	Total Sugar (%)	Total Acid (%, Citric Acid)	Soluble Solid (%)
***C. grandis* “Guanximiyu”**			
Dapu of Guangdong (GD)	10.46	1.16	12.08
Pinghe of Fujian (FJ)	10.90	0.66	11.97
Nanbu of Sichuan (SC)	11.25	1.06	12.40
***C. grandis* “Shatianyu”**			
Dapu of Guangdong (GD)	11.17	0.26	16.13
Pingle of Guangxi (GX)	11.12	0.31	13.10
Nanbu of Sichuan (SC)	9.13	0.45	10.20

### 4.3. Sample Preparation

The flavedo, the external layer of peel, and the flesh were separated from two symmetrical parts of the fruits. Afterwards, the flavedo or flesh of the fruits was combined and frozen using liquid nitrogen, and finally ground into a fine powder using a blender. The powder of the flavedo was stored at −40 °C before further analysis. The frozen flesh powder was thawed and then centrifuged at 8000 rpm for 10 min to collect the juice.

### 4.4. Flavonoid Extraction

The extraction of flavonoids from the flavedo and the juice of pummelo cultivar samples followed our published method [13]. Regarding the extraction of the flavonoids from the flavedo, the flavedo powder (15.0 g) was mixed with methanol (30 mL), sonicated for 5 min, and kept in the dark at 25 °C for 30 min with three agitations at 150 rpm. The resulting extract was centrifuged at 8000 rpm for 10 min, and the supernatant was collected. All the supernatants were pooled, evaporated at 30 °C on a rotary evaporator, and then redissolved in 10 mL of methanol prior to HPLC-MS analysis. Three fruits from the same tree were mixed as a replicate and extractions from three trees were considered triplicate tests. The extraction of the flavonoids from the juice was conducted by adding ethyl acetate (50 mL) to the juice (50 mL) five times. The extracts were pooled and evaporated on a rotary evaporator at 30 °C. Afterwards, the resultant residue was re-dissolved in 5 mL of methanol before HPLC-MS analysis. Three fruits from the same tree were mixed as a replicate and extractions from three trees were considered triplicate tests.

### 4.5. HPLC-MS/MS Analyses of Flavonoids

An Agilent 1100 system (Agilent Technologies, Santa Clara, CA, USA) consisting of an autosampler, a quaternary pump, a column compartment, and a diode array detector was used for separation of the flavonoids in the flavedo and the juice samples [13]. The separation was achieved using a reverse-phase Zorbax SB C18 column (250 mm × 4 mm, 5 µm, Agilent Technologies). The binary mobile phase consisted of (A) 1% acetic acid:water (v/v) and (B) 1% acetic acid:acetonitrile (v/v). A 32 min gradient was used as follows: 0 min, 5% B; 5 min, 8% B; 7 min, 12% B; 12 min, 18% B; 17 min, 22% B; 24 min, 35% B; 26 min, 100% B; 30 min, 100% B; 32 min, 5% B; followed by 5 min of re-equilibration of the column before the next run. The injection volume was 10 µL with a flow rate of 1.0 mL/min. The wavelength on the diode array detector was 280 nm. Electrospray ionization in negative mode was performed using nebulizer 30 psi, drying gas 10 L/min, drying temperature 325 °C, and capillary voltage 4000 V. Full scan mass spectra of the flavonoids were recorded from *m/z* 100 to *m/z* 1500. Naringin and quercitrin were used as external standards to quantify flavanones and flavones, respectively. Data were collected and integrated using Chemstation software (Agilent Technologies).

### 4.6. Statistical and Multivariate Analyses

Data were expressed as the mean ± standard deviation of triplicate tests. One-way analyses of variance of three-tree means were performed using SPSS Version 16.0 Statistical Package for Windows (SPSS Corporation, Chicago, IL, USA). A difference of *p* ≤ 0.05 was considered as significant. Cluster analysis was achieved by the same software using the individual flavonoids as variables.

## 5. Conclusions

In conclusion, the pattern and content of flavonoids in the flavedo and the juice of *C. grandis* “Guanximiyu” from the GD, FJ, and SC regions and of *C. grandis* “Shatianyu” from the GD, GX, and SC regions were compared using HPLC-MS/MS, respectively. The results showed that the accumulation of individual flavonoids was significantly affected by cultivation regions. Particularly, naringin, vicenin-2, and rhoifolin showed the highest content level in the flavedo and the juice of *C. grandis* “Guanximiyu” from the FJ region, the original cultivation region of *C. grandis* “Guanximiyu”. However, the original cultivation region for *C. grandis* “Shatianyu”, the GX region, had the lowest level of naringin and melitidin. The cluster analysis indicated that genotype determined flavonoid profiles, whereas cultivation regions affected flavonoid composition in pummelo cultivars.
